# Dental Department of the University of Maryland

**Published:** 1887-11

**Authors:** 


### Editorial, Etc.

---

Dental Department of the University of Maryland.—The class in this institution is as large at the time of writing as it was last year at the same period, notwithstanding the fact that no less than *five* new dental schools have been established during the present year. If the Association of Dental College Faculties would turn their attention to the matter of fees and insist that all colleges belonging to the Association charge uniform fees instead of the "cutting of rates," as it may be termed, which is now prevalent among a number of the lately organized schools, they would greatly benefit dental education.

